# A human carboxypeptidase E/NF-α1 gene mutation in an Alzheimer's disease patient leads to dementia and depression in mice

**DOI:** 10.1038/tp.2016.237

**Published:** 2016-12-06

**Authors:** Y Cheng, N X Cawley, T Yanik, S R K Murthy, C Liu, F Kasikci, D Abebe, Y P Loh

**Affiliations:** 1 Section on Cellular Neurobiology, Eunice Kennedy Shriver National Institute of Child Health and Human Development, National Institutes of Health, Bethesda, MD, USA; 2 Department of Biological Sciences, Middle East Technical University, Ankara, Turkey

## Abstract

Patients with Alzheimer's disease (AD), a common dementia among the aging population, often also suffer from depression. This comorbidity is poorly understood. Although most forms of AD are not genetically inherited, we have identified a new human mutation in the carboxypeptidase E (CPE)/neurotrophic factor-α1 (NF-α1) gene from an AD patient that caused memory deficit and depressive-like behavior in transgenic mice. This mutation consists of three adenosine inserts, introducing nine amino acids, including two glutamines into the mutant protein, herein called CPE-QQ. Expression of CPE-QQ in Neuro2a cells demonstrated that it was not secreted, but accumulated in the endoplasmic reticulum and was subsequently degraded by proteasomes. Expression of CPE-QQ in rat hippocampal neurons resulted in cell death, through increased ER stress and decreased expression of pro-survival protein, BCL-2. Transgenic mice expressing CPE-QQ did not show any difference in the processing enzyme activity of CPE compared with wild-type mice. However, the transgenic mice exhibited poor memory, depressive-like behavior, severely decreased dendrites in the hippocampal CA3 region and medial prefrontal cortex indicative of neurodegeneration, hyperphosphorylation of tau at Ser^396^, and diminished neurogenesis in the dentate gyrus at 50 weeks old. All these pathologies are associated with AD and the latter with depression and were observed in 50-week-old mice. Interestingly, the younger CPE-QQ mice (11 weeks old) did not show deficits in dendrite outgrowth and neurogenesis. This study has uncovered a human CPE/NF-α1 gene mutation that could lead to comorbidity of dementia and depression, emphasizing the importance of this gene in cognitive function.

## Introduction

Alzheimer's disease (AD) is a common form of dementia among the aging population and about 20–30% of AD patients also suffer from depression.^1^ Depression is the most common neuropsychiatric disorder associated with AD^2^ and is present primarily in the elderly.^3^ Studies have also shown an association between late-life depression and AD.^4^ Whether depression is a risk factor for AD or vice versa is unclear. Both AD and major depressive disorder (MDD) share an overlapping pathophysiology. The pathology of AD is typically characterized by: (1) the presence of neurofibrillary tangles (NFTs) formed by aggregates of hyperphosphorylated tau, a microtubule-associated protein in neurons,^5^ and (2) accumulation of abnormal peptides (Aβ_1–42_) proteolytically cleaved from β-amyloid precursor protein (APP), which form extracellular senile plaques.^6^ These NFTs and plaques, found primarily in the hippocampus and frontal cortex of the AD brain, trigger neurodegeneration in these areas. Increased hippocampal plaques and tangles have been found in AD patients that have a lifetime history of depression. Serum Aβ_1–40/42_ ratio was also higher in AD patients with depression.^7^ However, the etiology of the comorbidity of depression and cognitive/memory deficits in AD is not well understood. Whether there might be certain forms of AD arising from specific genetic mutations that predispose individuals to both dementia and depression needs to be explored.

Most forms of AD are not genetically inherited. Only <0.3% of AD cases are autosomal dominant familial AD (not sex-linked) and the onset of the disease occurs early, before age 65.^8, 9^ Autosomal dominant familial AD is attributed to mutations in one of three genes: *APP*, *presenilins 1* and *2.*^10, 11, 12^ Mutations in these genes generally lead to an increase in the production of Aβ_1–42_, the major component of senile plaques.^13^ More recently, high throughput genetic analyses have identified many single nucleotide polymorphisms (SNPs) associated with increased risk factors for AD, rendering the possibility of a greater genetic contribution to AD. Genome-wide association studies have identified some of these genes as *apolipoprotein E* (*APOE*), *TOMM40,* and a hypothetical gene *LOC100129500* partially overlapping with APOE, as well as a novel gene, *EPC2*.^14^ For the *APOE* gene, inheritance of the ɛ4 allele increases the risk of AD by 3 times in heterozygotes and 15 times in homozygotes.^9^ In addition, nine other genes with AD risk SNPs (*CLU, CR1, BINI1, ABCA7, MS4A, CD33, CD2AP, EPHA1* and *TREM2*) have been reported.^15^ Systematic meta-analyses of AD genetic association studies have also identified many potential AD susceptibility gene candidates.^16^ However, no studies have demonstrated that any of these genes are associated with depression.

In exploring the possibility of a genetic component for comorbidity of dementia and depression in AD, we focused on previous studies on the carboxypeptidase E (CPE)/neurotrophic factor-α1 (NF-α1) knockout mouse model, which showed both severe neurodegeneration^17^ and depression.^18^ A search of various databases uncovered a novel mutation in the *CPE/(NF-α1)* gene in an expressed sequence tag (EST) reported by the Helix Research Institute, Japan, obtained from the cortex of an AD patient.^19^ CPE, first identified as a prohormone processing enzyme,^20, 21^ is located on chromosome 4 at 4q32.3 in the human genome.^22^ CPE is expressed during early embryonic development in rats.^23^ It is present in neurons and glial cells^24^ and has also been demonstrated to be a neuroprotective protein.^17, 25^ Knockout mice, lacking CPE/NF-α1, exhibit obesity, infertility and type 2 diabetes, reflective of the lack of or reduced levels of bioactive peptides.^24, 26^ In addition, they exhibit severe neurodegeneration in the CA3 region of the hippocampus and have poor cognitive function, including deficits in learning and memory.^17^ CPE/NF-α1 has been shown to act extracellularly, through the ERK and Akt signaling pathways to enhance expression of BCL-2, a pro-survival protein, to mediate neuroprotection.^27^ These CPE/NF-α1 knockout mice also showed diminished neurogenesis in the dentate gyrus of the hippocampus and depressive-like behavior, which was rescued by subcutaneous administration of FGF2, which induced neurogenesis.^18^ Thus, the mutation in the *CPE* gene found in the AD patient could potentially contribute to the histopathology, memory deficits and depression associated with AD.

**Figure 1:**
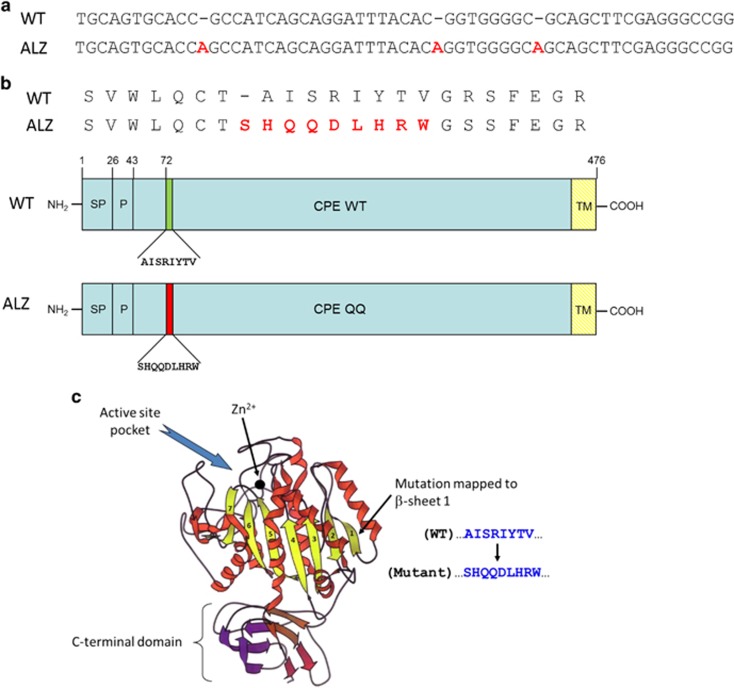
Nucleotide and amino acid sequence of human wild-type (WT) and Alzheimer's disease (AD) mutant carboxypeptidase E (CPE). (**a**) Inserted nucleotides in the mutant CPE sequence obtained from the human EST sequence of Alzheimer's patient, DA134138.1 (RNA from Invitrogen, Cat. #D6830-01) is shown in red. (**b**) Amino acid alignment of human WT-CPE and the mutant CPE, the inserted amino acids are shown in red. The schematic diagram shows the linear domain, where the nine amino acids are introduced in the mutant protein. As a pair of QQ amino acids is introduced, the mutant will be referred to as CPE-QQ. (**c**) X-ray crystal structure of the catalytic domain II of duck carboxypeptidase D (CPD, Protein Data Bank ID #1qmu), a homologous protein to CPE. The QQ mutation occurs in the first β-sheet of CPD (see arrow), which is associated in an anti-parallel manner with the β-sheet 2.

The *CPE* mutation found in the AD patient contains three adenosine nucleotide insertions that result in the substitution of amino acids after the pro-domain of the enzyme (Figure 1). The mutation results in nine new amino acids replacing eight original amino acids at the N terminus in the mutant protein compared with the wild-type (WT) CPE. The new amino acids added contain two adjacent glutamine (Q) residues; hence, we termed it a CPE-QQ mutant. Interestingly, another analysis of polyA+ RNA pooled from thalami of humans that died from brain trauma uncovered an EST sequence with a similar mutation that resulted in a predicted CPE protein sequence that contained the addition of the same nine amino acids as the AD patient (Supplementary Figure 1).

In the present study, we have characterized this CPE-QQ mutant protein found in the AD patient, and examined the possibility that it could give rise to the pathologies and behavioral deficits characteristic of AD and depression. Indeed, we found that this CPE-QQ mutant when expressed in hippocampal neurons led to cell death, mediated by a mechanism involving endoplasmic reticulum (ER) stress. Furthermore, transgenic mice expressing this mutant exhibited memory deficits, depressive-like behavior, neuronal degeneration, increased tau phosphorylation and decreased neurogenesis in the hippocampus. Our findings indicate that this human CPE mutation could result in neurodegeneration, memory deficits and depression linked to AD.

## Materials and methods

### Bioinformatic search for *CPE* gene mutation

A bioinformatic strategy was designed to find posttranscriptional modifications in the CPE coding region, such as alternative splicing events. Using CPE protein sequence (NCBI Reference Sequence: NP_001864), a TBLASTN (search translated nucleotide databases using a protein query) search was performed on non-redundant nucleotide sequence database. Threshold were set to return maximum target hits, with an expected threshold of ‘10'. Scoring parameters were set to ‘ BLOSUM62' matrix with a Gap costs of ‘Existence: 11 Extension:1'. Filters were set to avoid ‘Low complexity regions'. Among several thousand hits, specific sequence alignments were manually selected that have Gaps, positive amino acids or split in the amino acid sequence alignment but still following the same frame of the CPE open reading frame (ORF). Using this search strategy, we identified an EST |DA134138.1| that had three adenosine inserts that introduces nine amino acids at the N terminus of the CPE protein but still maintaining the ORF. Owing to the presence of two glutamine residues in the new sequence it was called CPE-QQ sequence. Following EST accession # DA134138.1, we found that it was one of the collections of FLJ Human cDNA Database (http://flj.lifesciencedb.jp/top/) and DA134138.1 was from cortex RNA of a 65-year-old male AD patient sequenced for a high throughput studies to identify alternative promoter regions of human genes among normal and diseased patients.

### Generation of the CPE-QQ construct and transgenic mouse

A construct containing the ORF of human CPE-QQ was custom synthesized and cloned into the pcDNA3.1(+) expression vector with *BamH*1/*Xho*1 cloning sites (GenScript, Piscataway, NJ, USA). Sequence analysis confirmed the insertion of three adenosine nucleotides consistent with the EST database sequence of the mutant hCPE (Genbank ID# DA134138). The pcDNA3.1 vector was digested with *Nru*I/*Pvu*II to generate a 2.44 kb fragment containing the CMV enhancer and promoter, the CPE-QQ ORF and the vector poly A site. This fragment was purified and used for transgenic mouse production by Dr James Pickle (Transgenic Core Facility, NIMH, NIH) on a C57/BL6 background. Founder animals were genotyped with two sets of primers; QQ1 5′-CCGCGTTACATAACTTACGGTAAATGGCC-3′, QQ2 5′-GGGTCTCCCTATAGTGAGTCGTATTAATTTCG-3′ that amplifies the 5′-upstream sequence of the pcDNA3.1 vector only; and QQ4 5′-GCTTGCTCCTGAGACCAAGGCTG-3′, QQ6 5′-GCCTGCTATTGTCTTCCCAATCCTCC-3′ that amplifies the 3′-end of hCPE and the downstream pcDNA3.1 vector. Positive founders were mated with C57/BL6 WT mice to propagate the lines, and F1 and subsequent generations were genotyped for the transgene identification. All animal studies were done in accordance with the NICHD Animal Care and Use Committee guidelines.

### Cell biological analysis of Neuro2a and COS-7 cells expressing CPE-QQ

#### Cells

Neuro2a cells (N2a), a mouse neuroblastoma cell line and COS-7 cells, an African green monkey kidney cell line, were purchased from ATCC (Manassas, VA, USA) and cultured at 37 °C under an atmosphere of 5% CO_2_ in Dulbecco's modified Eagles medium (DMEM) supplemented with 10% fetal calf serum, 100 units ml^−1^ penicillin, 100 μg ml^−1^ streptomycin and 4 mm glutamine. The cells were grown to ~70% confluency and transiently transfected with CPE-WT, CPE-QQ, a mixture of both constructs where noted or empty vector (EV) for 24 h using Lipofectamine 2000 (Invitrogen, Carlsbad, CA, USA) according to the manufacturer's protocol.

#### Enzymatic activity assays

COS-7 cells, transfected with CPE-WT or CPE-QQ, as described above, were harvested in 50 mm sodium acetate, pH 5.5, 0.01% Tx-100 and phenylmethylsulfonyl fluoride (1 mm), and frozen and thawed once. The samples were centrifuged at 13 000 r.p.m. for 20 min in a microfuge and the supernatant saved. Aliquots were made and frozen at −80 °C until use. The COS-7 lysates containing ~100 ng protein were incubated for 18 h at 37 °C in 50 mm sodium acetate, pH 5.5 with 5 μg adrenocorticotropic hormone (ACTH) (1–17) peptide, with and without cobalt (1 mm) and/or the CPE inhibitor, GEMSA (5 μm). The peptides were separated by HPLC on a 4.6 × 250 mm 5 μm reverse phase Jupiter C18 column (Phenomenex, Torrance, CA, USA) and detected by absorbance at 214 nm. Buffer A was 0.1% TFA and buffer B was 80% acetonitrile/0.1% TFA and the gradient was 30–40% B in 12 min. Western blot analysis confirmed equivalent levels of CPE-WT and CPE-QQ protein in the cell extracts (data not shown).

#### Secretion assays

For N2a cell secretion experiments, cells expressing CPE-WT, CPE-QQ or both CPE-WT/CPE-QQ, were washed with DMEM and then incubated with DMEM supplemented with 0.01% BSA for 5 h at 37 °C. The media were collected and precipitated with 10% ice cold tri-chloroacetic acid and the recovered proteins analyzed by western blot. The cells were lysed with M-PER lysis buffer (Thermo Scientific, Waltham, MA, USA), supplemented with 1 mm phenylmethylsulfonyl fluoride inhibitor. The cell lysate was centrifuged at 13 000 *g* for 10 min at 4 °C and the supernatant collected for analysis by western blot as follows. N2a cell lysate and 20 μl of concentrated media were denatured, run on 4–20% sodium dodecyl sulfate–polyacrylamide gel electrophoresis gels and transferred onto nitrocellulose membrane. The CPE-WT and CPE-QQ were detected using mouse anti-CPE monoclonal antibody (1:5000, BD Biosciences, San Jose, CA, USA) followed by IRDye conjugated secondary antibodies (Rockland Immunochemicals, Gilbertsville, PA, USA) and analyzed with an Odyssey Infrared Imager System and application software (LI-COR Biosciences, Lincoln, NE, USA).

#### Inhibitor studies

For inhibitor studies on the degradation of CPE-QQ and CPE-WT, equivalent amounts of N2a cells were stably transfected with human CPE-WT, CPE-QQ or EV (pcDNA3.1, negative control) cDNAs. Cells were then treated with a proteasomal inhibitor, MG132 (5 μm, Sigma, St Louis, MO, USA) or a lysosomal inhibitor, E64d (20 μm, Sigma), for 24 h and then collected for western blot analysis. The cells were extracted with M-PER lysis buffer, quantified by the Bradford Assay (Thermo Scientific) and equal protein amounts were analyzed by western blot with a mouse anti-CPE monoclonal antibody (1:10 000, R&D Systems, Minneapolis, MN, USA) and then with a mouse anti-β-actin monoclonal (1:2000, Cell Signaling, Danvers, MA, USA) antibody. The bands were visualized with luminal:peroxide ECL substrate (Thermo Scientific). A Kodak X-Ray processor was used for fixing and developing the signals on the X-ray films. The bands were quantified by using Fuji (Image J) program (background subtracted). In similar experiments, cell extracts were analyzed by western blot for levels of CHOP, an ER stress-induced transcription factor.

#### Immunocytochemical analysis

N2a cells expressing CPE-WT and CPE-QQ proteins were processed for immunocytochemistry (ICC) as previously described^28^ and immunostained for CPE using mouse anti-CPE monoclonal antibody (1:400, BD Biosciences) and rabbit anti-Calnexin (1:1000, Sigma). Secondary antibodies were anti-mouse-Alexa488 and anti-rabbit Alexa568 at 1:1000 each (Thermo Fisher Scientific, formerly Molecular Probes). The stained cells were visualized on a 510 Inverted META confocal microscope.

### Cell biological analysis on primary neuronal cultures expressing CPE-QQ

#### Cells

Hippocampal neuronal cultures were prepared as described previously.^27^ The neurons were infected (multiplicity of infection 150) with adenovirus expressing β-galactosidase (LacZ), CPE-WT or CPE-QQ (Vector Biolabs, Malvern, PA, USA) for 96 h. After this time, various experiments were performed to measure the effects of transduction. For rescue experiments, the neurons were treated with recombinant NF-α1/CPE (0.4 μm) or transduced with adenovirus expressing Bcl-2 (multiplicity of infection 20) for a further 96 h. In secretion experiments, media from 1 × 10^6^ hippocampal neurons grown in 1 ml of culture medium (Neurobasal+2% B27) were collected, centrifuged for 15 min at 12 000*g* to remove cell debris and the supernatant analyzed by western blot.

#### WST-1 assay for cell viability

The viability of the cells was determined by the WST-1 Cell Proliferation Reagent (Clontech, Mountain View, CA, USA) assay in a 96 well plate according to the manufacturer's protocol. Briefly, after the different treatments, 10 μl of premixed WST-1 was added to each well and the plate was maintained in a 37 °C incubator for 1–2 h. The absorbance of the samples was then measured at 440 nm using a multi-well plate reader.

#### Lactate dehydrogenase release assay for cell cytotoxicity

The cytotoxicity of the cells after various treatments was evaluated by the extent of the release of lactate dehydrogenase. This was achieved with a CytoTox 96 Non-Radioactive Cytotoxicity Assay kit according to the manufacturer's instructions (Promega, Madison, WI, USA).

#### Western blotting

Protein lysates of hippocampal tissue and hippocampal neurons in culture were prepared by homogenizing with T-protein extraction reagent (T-PER, Thermo Scientific), supplemented with Complete Inhibitor Cocktail (Roche, Indianapolis, IN, USA). The lysates were collected, centrifuged at 13 000 *g* for 10 min at 4 °C and the protein concentrations from supernatants determined. Twenty micrograms of protein were denatured at 90 °C for 3 min and run on 4–20 or 12% sodium dodecyl sulfate–polyacrylamide gel electrophoresis gels and transferred onto nitrocellulose membrane (Millipore, Billerica, MA, USA), according to the standard protocol. After blocking with 5% nonfat milk at room temperature for 1 h, CPE on the membrane was detected using a mouse CPE monoclonal antibody directed against the 49–200 amino acid sequence (BD Biosciences) at 1:5000. BCL-2 was detected using rabbit anti-BCL-2 monoclonal antibody (Cell Signaling) at 1:2000. Phosphorylated tau was detected by polyclonal rabbit anti-p-tau^Ser396^ (1:3000, Santa Cruz, Dallas, TX, USA) antibody. CCAAT-enhancer-binding protein homologous protein (CHOP) was detected by monoclonal mouse anti-CHOP antibody at 1:3000 (Cell Signaling). Following primary antibody binding, the membrane was incubated with fluorescent conjugated anti-mouse or rabbit antibodies (Amersham, Piscataway, NJ, USA) and visualized by the Odyssey infrared imaging systems version 2.1 (LI-COR, Lincoln, NE, USA) and bands were quantified by Odyssey software and expressed as arbitrary units (AU). The CPE expression level for each sample was normalized with β-actin and expressed as the mean±s.e.m. of AU from three separate experiments.

### Analysis of CPE-QQ transgenic mice

#### Immunohistochemistry

The brains of 50-week-old WT and CPE-QQ transgenic mice were dissected from perfused mice and embedded in a gelatin matrix using MultiBrain Technology (NeuroScience Associates, Knoxville, TN, USA) or each brain was embedded and sectioned, all the sections were coronal. The MultiBrain block was sectioned in the coronal plane at 35 μm on an AO 860 sliding microtome. The sections were then stained for doublecortin (DCX), p-tau, or MAP2 using polyclonal rabbit anti-doublecortin (1:2000, Cell Signaling), polyclonal rabbit anti-p-tau^Ser396^ (1:1000, Santa Cruz), and monoclonal mouse anti MAP2 antibody (1:3000, Abcam, Cambridge, MA, USA), respectively, followed by Alexa Fluor 594 goat anti-rabbit secondary antibody (Invitrogen). After mounting of the sections, images were taken from the hippocampus by a confocal microscope (Zeiss LSM 510 Inverted Meta, Carl Zeiss Microscopy, Thornwood, NY, USA) with × 20 or × 63 magnification. For counting of DCX immunoreactive cells in the dentate gyrus, a fluorescent microscope (Nikon eclipse 80i, Nikon Instruments, Melville, NY, USA) with a digital camera was used (× 20 objective).

#### Body weight and blood glucose measurements in transgenic mice

Fifty-week-old WT and CPE-QQ transgenic mice were weighed at 0900 hours and blood glucose was measured with a glucometer.

#### Behavioral studies

The Morris Water Maze was used to test spatial learning and memory. Testing was performed in a circular pool, 1.2 m in diameter, filled with water made opaque with the addition of nontoxic white paint. Videotracking was achieved with the Anymaze software (Stoerlting, Wood Dale, IL, USA) connected to a video camera and navigational parameters were analyzed subsequently with the Anymaze software. To measure the animals' learning ability, a submerged platform was placed in one quadrant of the pool. On day 0, the mice were allowed to acclimatize in the apparatus as follows: the mouse was put on the platform for 10 s, then let swim for 20–30 s, and then guided to the platform for 10 s. On day 1, animals were placed into the pool, facing the wall of the pool, in a new quadrant on each of the successive four trials per day. The hidden platform remained in the same position for all these trials. Mice were allowed a maximum of 90 s to locate the platform and were left on the platform for 30 s before being removed. If the platform was not reached in 90 s, the mouse was guided to the platform and allowed to sit on it for 30 s. Escape latency was measured for five consecutive days for the animals' learning ability test. Twenty-four h after the fifth day, a probe test was performed to measure the memory of the animals. The hidden platform was removed from the pool, and the animals were placed at the opposite quadrant where the hidden platform used to be located. The mice were allowed to explore the pool for 90 s and the activity of the mice was recorded and analyzed by the Anymaze software. Animals were excluded from analysis if they did not search the platform to escape.

The forced swim test was used to test for depressive-like behavior. The forced swim test was conducted at 1300–1700 hour similarly to that previously described.^18^ Briefly, the apparatus consisted of a 4-litre Pyrex beaker (15 cm diameter) that contained 15 cm water (25 °C). Swimming/immobility behavior was digitally recorded for 6 min and scored using the Anymaze software. Immobility time is defined as the time that the animal spent floating or engaged in minimal activity to keep afloat. WT and litter-matched CPE-QQ mice 30–40 weeks of age were allowed to swim/struggle/float for 6 min before being returned to the home cage. Fresh water was used for each mouse. The data are presented as means and standard errors of the mean.

Sucrose preference test was performed as previously described.^18^ Briefly, the mice were habituated to the presence of two bottles containing sterile water for 5 days. Following this acclimation, mice had the free choice of either drinking the 1% sucrose solution or sterile water for a period of 4 days. Sucrose preference was calculated as a percentage of the volume of sucrose intake over the total volume.

All animals were male and randomly selected for experiments at certain ages in this study. The experimenter was blinded to the group allocation during animal experiments, and was given the code by another investigator to analyze the data after experiments.

#### Statistical analysis

Data were analyzed by student *t*-test or one-way analysis of variance (ANOVA) followed by Tukey *post-hoc* multiple comparisons tests where noted. Data are presented as means plus/minus standard error of mean. Significance was set at *P*<0.05.

## Results

### Bioinformatic analysis identifies *CPE* gene mutation in AD patient

A non-redundant nucleotide sequence database search with the human *CPE* nucleotide sequence as queries (NM_001873.2 and NM_013494.3, respectively) against the GeneBank EST database identified an EST sequence entry from cortex RNA of a 65-year-old male AD patient^19^ that had three adenosine inserts (for details of bioinformatics methodology used, see ‘Materials and methods' section, Figure 1a). This introduces nine new amino acids in the mutant protein, replacing eight amino acids in CPE-WT (Figure 1b) in the first beta-pleated sheet region after the pro-domain of the CPE protein (Figure 1c), herein called CPE-QQ due to the presence of two glutamine residues in the new sequence (Figures 1b and c).

### Transgenic mice expressing CPE-QQ exhibit memory deficits and depressive-like behavior

**Figure 2:**
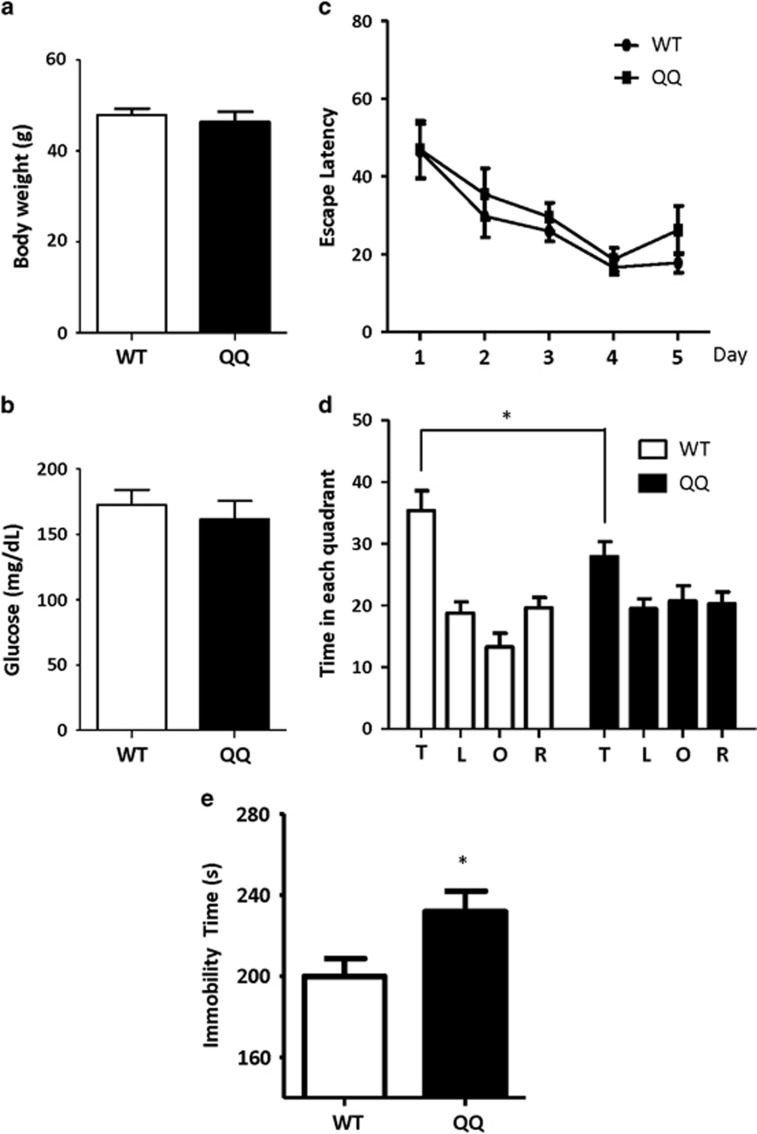
Carboxypeptidase E (CPE)-QQ transgenic mice exhibit memory deficits in Morris water maze test. (**a**, **b**) Bar graphs show no difference in body weight (**a**) and fasting glucose levels (**b**) between wild-type (WT) and CPE-QQ mice. The values are the mean±s.e.m., *t*-test, *P*>0.05, *n*=8. (**c**) Fifty-week-old CPE-QQ mice were subjected to the Morris water maze test. Graph shows the latency escape over 5 days. WT and QQ mice exhibited a normal acquisition curve in the water maze task, suggesting that the transgenic mice had normal learning ability. Two way ANOVA of genotype: F_(1,113)_=1.627, *P*>0.05. *n* value: WT=12, CPE-QQ=11. (**d**) Bar graphs show time spent by the mice in each quadrant during probe test. WT mice spent almost twice as much time in the target quadrant, whereas QQ mice did not, indicating that the transgenic mice have spatial memory deficits. (T: target quadrant, L: left quadrant, O: opposite quadrant, R: right quadrant.) *t*-test of target quadrant: *P<0.05. *n* value: WT=12, CPE-QQ=11. (**e**) CPE-QQ mice spent more time immobile in the forced swim test than the WT mice, indicative of depressive-like behavior. *t*-test, *P<0.05, *n* value: WT=11, CPE-QQ=13.

To explore the biological function of the mutation, we made transgenic mice expressing the CPE-QQ mutation. Although the ratio of WT:QQ protein expressed could not be quantified due to lack of a specific antibody to the CPE-QQ protein, mRNA expression levels determined by RT-PCR showed CPE-QQ mRNA expression was only found in the transgenic mice and there was no difference in WT-CPE mRNA levels (Supplementary Figure S1). We also examined body weight and fasting blood glucose levels in the CPE-QQ mice as the CPE-knockout mice show these metabolic changes.^26^ Figures 2a and b show that CPE-QQ mice did not exhibit any difference in body weight and fasting blood glucose levels compared with WT animals, respectively.

The animals were tested for their learning and memory deficits using the Morris Water maze test. There were no differences in the rate of acquisition of learning ability (Figure 2c) or swim speed (Supplementary Figure S2) between the CPE-QQ and WT animals at 50 weeks. The learning ability was also not different between genotypes at 90 weeks (Supplementary Figure S3A) of age. Spatial memory consolidation is recorded as the time the mouse spends in each quadrant during the probe test following the escape latency test. Both 50- (Figure 2d) and 90-week-old (Supplementary Figure S3B) CPE-QQ mice spent less time in the target quadrant compared with age-matched controls. In addition, we analyzed the difference between the target quadrant and other quadrants per strain in 50-week-old mice. In general, both CPE-QQ and WT mice spent more time in the target quadrant compared with the other quadrants; however a greater significant difference between target quadrant and other quadrants were found in WT mice (For WT mice: T vs L, *P*<0.001; T vs O, *P*<0.001; T vs R, *P*<0.001. For CPE-QQ mice: T vs L, *P*=0.008; T vs O, *P*=0.048; T vs R, *P*=0.02. *t*-test). These results indicated that these CPE-QQ mice had deficits in spatial memory.

We next tested for depressive-like behavior of the animals by the forced swim test. CPE-QQ mice had a significantly increased immobility time compared with WT mice in the forced swim test (Figure 2e), indicating that the CPE-QQ mice had depressive-like behavior. However, the CPE-QQ mice showed similar sucrose preference as the WT mice (Supplementary Figure S4), this may be due to the milder depression in CPE-QQ mice compared with CPE KO mice.

### Transgenic CPE-QQ mice show decreased dendrites, phosphorylated tau and decreased neurogenesis in the hippocampus

**Figure 3:**
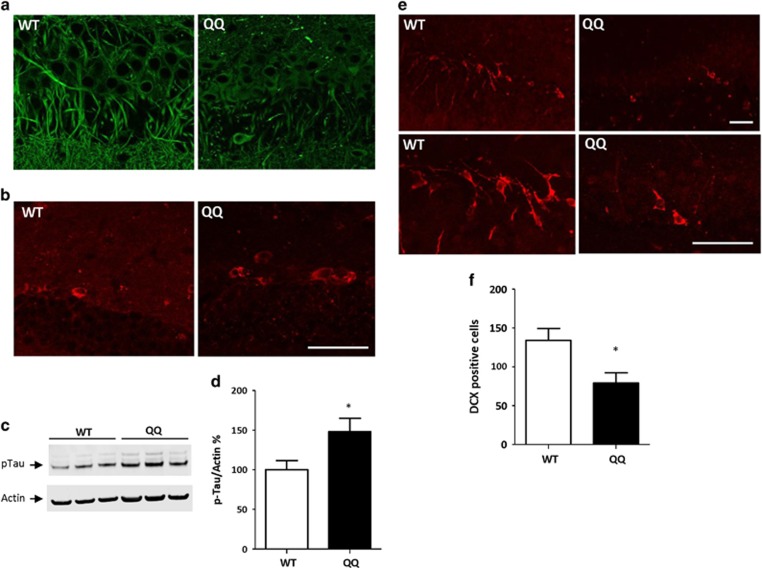
Histopathology of carboxypeptidase E (CPE)-QQ mouse brain. (**a**) Confocal images (× 63) showing much fewer dendrites indicating neurodegeneration in CPE-QQ mice (50 weeks old) compared with the wild-type (WT) mice in CA3 region of hippocampus, in CPE-QQ mice. (**b**) Confocal images (× 63) showing phosphorylation of tau in DG of hippocampus in WT and QQ mice. (**c**, **d**) Western blot analysis of p-Tau levels in hippocampus. Note that 50-week-old QQ mice had more tau phosphorylation in hippocampus than WT mice. *t*-test, *n*=6, *P<0.05. (**e**) Top panels: Confocal images (× 20) and bottom panels of confocal images (× 63) showed that 50-week-old QQ mice had less double cortin staining in the dentate gyrus (DG) compared with WT mice, indicative of less neurogenesis in QQ mice. (**f**) Quantification of doublecortin positive cells in DG. *t*-test, *n*=4, *P<0.05. For all confocal images, scale bar: 50 μm. *n* value: WT=4, CPE-QQ=4.

To examine the histopathology of the CPE-QQ mouse brain, we carried out immunohistochemistry on 50-week-old mice. Nissl staining showed that there was no apparent neuronal loss in the CPE-QQ mice compared with the WT mice in the hippocampus (Supplementary Figure 5A). However, when the sections were then stained with microtubule-associated protein 2 (MAP2), a specific neuronal marker, the results show that there were much fewer dendrites in the CA3 region and dentate gyrus of the hippocampus of CPE-QQ mice compared with WT controls (Figure 3a and Supplementary Figure 5B). Moreover, we also found fewer dendrites in the prefrontal cortex of the CPE-QQ mice (Supplementary Figure 5C). Our study showed that phosphorylation of tau using an antibody specific for Ser^396^, that is well documented in AD cases,^29^ was significantly increased in the dentate gyrus of the CPE-QQ mice compared with control mice (Figures 3b–d). We also examined doublecortin staining, a marker for immature neurons in the dentate gyrus of CPE-QQ mice. The number of doublecortin positive cells present was significantly decreased by ~50% compared with control WT mice, indicating less neurogenesis in the mutant mice (Figures 3e and f). These results taken together indicate that the CPE-QQ mice have many of the histopathological characteristics of dementia, including AD, as well as diminished neurogenesis characteristic of depression. We also carried out immunohistochemistry on 11-week-old mice. MAP2 staining showed a similar pattern of dendrite outgrowth in the dentate gyrus of the hippocampus of CPE-QQ mice compared with WT mice (Supplementary Figure S6A); and doublecortin staining suggested that the younger CPE-QQ mice did not have obvious deficits in hippocampal neurogenesis (Supplementary Figures S6B and C).

### CPE-QQ protein lacks enzymatic activity, is not secreted and induces ER stress in Neuro2a cells

**Figure 4:**
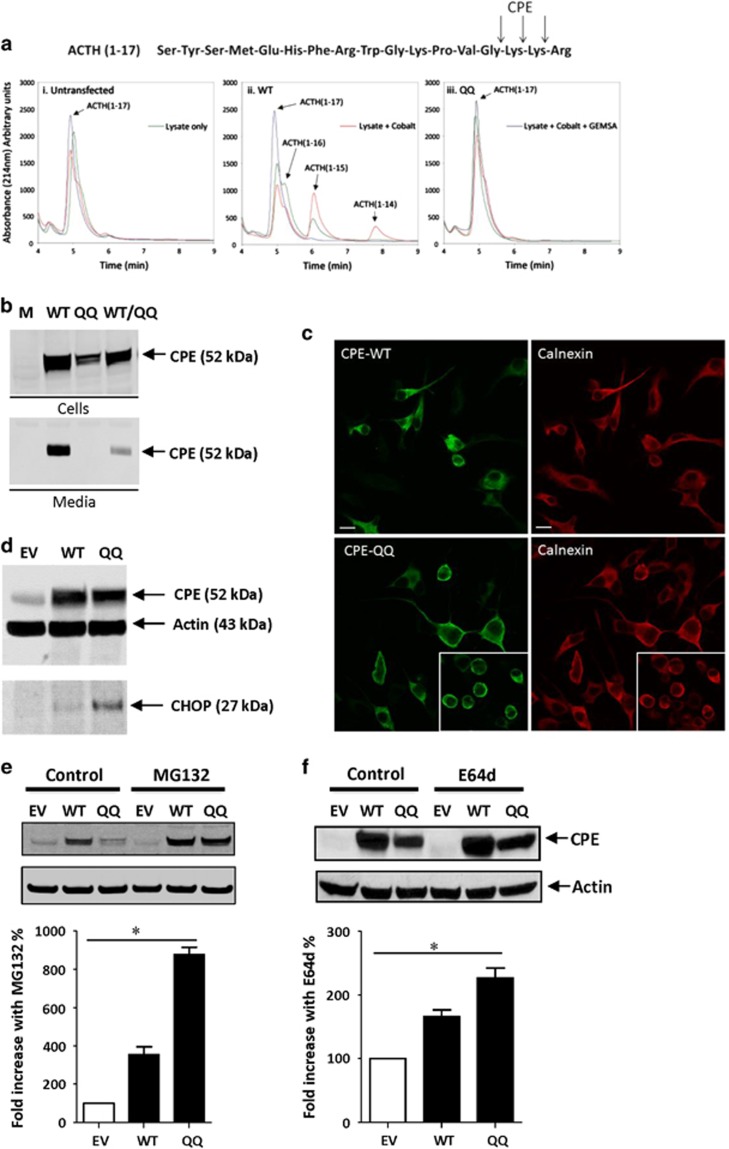
Effects of carboxypeptidase E (CPE)-QQ mutation on its enzyme activity and on cell viability. (**a**) Untransfected COS-7 cells (i) or cells expressing CPE-wild-type (WT) (ii) and CPE-QQ (iii) were extracted. The lysates were incubated for 18 h with ACTH(1–17) with and without cobalt and/or GEMSA and the enzymatically processed products were analyzed by HPLC. Note that only CPE-WT generated the appropriate peptide products (ACTH (1-16), ACTH (1-15) and ACTH (1-14) in a cobalt inducible and GEMSA inhibitable manner. (**b**) Western blot analysis of the cell lysate and media from Neuro2a cells. Cells were transfected with CPE-WT (WT), CPE-QQ (QQ) or empty vector (EV). Note that there was a decreased amount of CPE-QQ compared with WT in cells and none was secreted into the media, whereas the CPE-WT was (bottom panel). Also, note that when CPE-QQ and WT were expressed together, there was a significant reduction of CPE-WT in the media (21.6±6.8% compared with WT alone (100%), *n*=5). (**c**) Immunofluorescence confocal microscopy of Neuro2a cells expressing WT and CPE-QQ. Double immunostaining of WT-CPE (left panel) or CPE-QQ (right panel) (green) with Calnexin (red) in Neuro2a cells. CPE-QQ is localized in the perinuclear area and in a reticular distribution consistent with its localization in the endoplasmic reticulum (ER) in these cells. Note that cells highly expressing the QQ CPE mutant appear to be more rounded indicative of unhealthy cells (insert). (**d**) Western blot of CHOP in Neuro2a cells. Representative blot from two experiments are shown. Upper panel: cells were transfected with empty vector (EV), wild-type CPE (WT) and the CPE mutant (QQ). Lower panel: CHOP levels increased in cells expressing CPE-QQ compared with WT. (**e**, **f**) Rescue of degradation of CPE-QQ by proteosomal and lysosomal enzyme system inhibitors. Neuro2a cells were transfected with CPE-WT (WT), CPE-QQ (QQ) or EV, and treated with and without MG132, a proteosomal inhibitor (**e**) or E64d (**f**), a lysosomal inhibitor. Representative blots of three experiments are shown. Bar graphs show fold increase (%) of CPE protein, normalized against actin, in treated versus untreated cells expressing CPE-WT (WT), CPE-QQ (QQ) and endogenous CPE-WT (EV). The values are the mean±s.e.m., one-way ANOVA analysis ((**e**) (MG132): F(2,9)=147.9, *P*<0.001; (**f**) (E64d): F(2,6)=33.87, *P*<0.001), followed by Tukey test, *P<0.05 compared with the EV (control) group.

Next, we investigated the cell biological mechanism underlying how CPE-QQ could cause neurodegeneration using *ex vivo* systems. The additional new amino acids in the mutant have a calculated isoelectric point (pI) of 6.9, in contrast to the WT sequence that has a pI of 8.8. This change in pI, would predict a significant effect on the structure of the protein that may affect its enzymatic activity. To investigate the enzymatic activity, plasmids expressing CPE-QQ and CPE-WT, were transfected into COS-7 cells, which normally do not express CPE, and soluble cell extracts were made. Two preliminary analyses using ACTH(1–17) peptide as substrate (see Figure 4a for sequence), were carried out to initially demonstrate CPE activity in the WT and not the QQ expressing cells. A third, more comprehensive analysis is described here. Soluble cell extracts were incubated with ACTH(1–17), with and without cobalt, which stimulates CPE enzymatic activity, and/or the CPE inhibitor, GEMSA (Figure 4a). Lysates containing WT-CPE generated peptide products: ACTH(1-16), (1-15) and (1-14) representing sequential C-terminal cleavages of the basic residues of the substrate, in a cobalt inducible and GEMSA inhibitable manner (Figure 4a, middle panel). In contrast, lysates containing CPE-QQ did not generate any cleavage products (Figure 4a, right panel), similar to the control lysate from untransfected cells that contain no CPE (Figure 4a, left panel). This result indicates that CPE-QQ is enzymatically inactive.

To investigate if CPE-QQ is correctly trafficked in the cell, we examined the secretion behavior of CPE-QQ from N2a cells, a mouse neuroblastoma cell line, compared with CPE-WT. Western blot analysis (Figure 4b) shows that although the CPE-QQ was expressed (top panel), there was no secretion of the mutant in the medium (bottom panel), compared with WT-CPE, which was secreted. This observation suggests that CPE-QQ may not be trafficked through the secretory pathway, but may be trapped in the ER. Interestingly, when CPE-QQ was co-expressed with WT, secretion of the WT was reduced to 21.6±6.8% (±s.e.m., *n*=5) compared with cells expressing CPE-WT alone (right panel), suggesting that WT-CPE might co-aggregate with CPE-QQ in the ER, and is inhibited from traversing the secretory pathway.

To study the cellular localization of CPE-QQ in N2a cells, we carried out ICC studies of N2a cells transfected with CPE-QQ or CPE-WT. Figure 4c shows that CPE-QQ stains in the perinuclear region of the cells and co-stained with Calnexin, an ER marker, consistent with localization of the mutant in the ER (Figure 4c, lower left and right panels). Moreover, many CPE-QQ cells appear rounded (Figure 4c, lower panels, insert), indicating unhealthy cells that might be undergoing ER stress, unlike the cells expressing WT, which show staining in the cell body and neurites (Figure 4c, upper panels). To determine if cells expressing CPE-QQ are undergoing ER stress, we carried out western blot analysis using the anti-CHOP antibody. The ER stress marker, CHOP, was significantly elevated in N2a cells expressing CPE-QQ compared with those expressing CPE-WT or EV control (Figure 4d, *n*=2).

As the CPE-QQ protein appears to be accumulated in the ER causing ER stress we examined the degradation systems that might be responsible for degrading the mutant protein. Figure 4e shows that the proteasome inhibitor, MG132, significantly inhibited the degradation of CPE-QQ showing an approximately eightfold higher accumulation of the protein in the cell on treatment with the inhibitor. We then examined whether the cell permeable lysosomal inhibitor, E64d (thiol protease inhibitor) could inhibit the degradation of CPE-QQ. E64d treatment inhibited degradation of CPE-QQ, showing a approximately twofold increase in accumulation in the cell (Figure 4f). These results suggest that degradation of CPE is primarily through the proteosomal system, although some degradation may be occurring through autophagy from the ER and final degradation when autophagic bodies fuse with lysosomes.^30^

### CPE-QQ causes cytotoxicity through ER stress in hippocampal neurons

**Figure 5:**
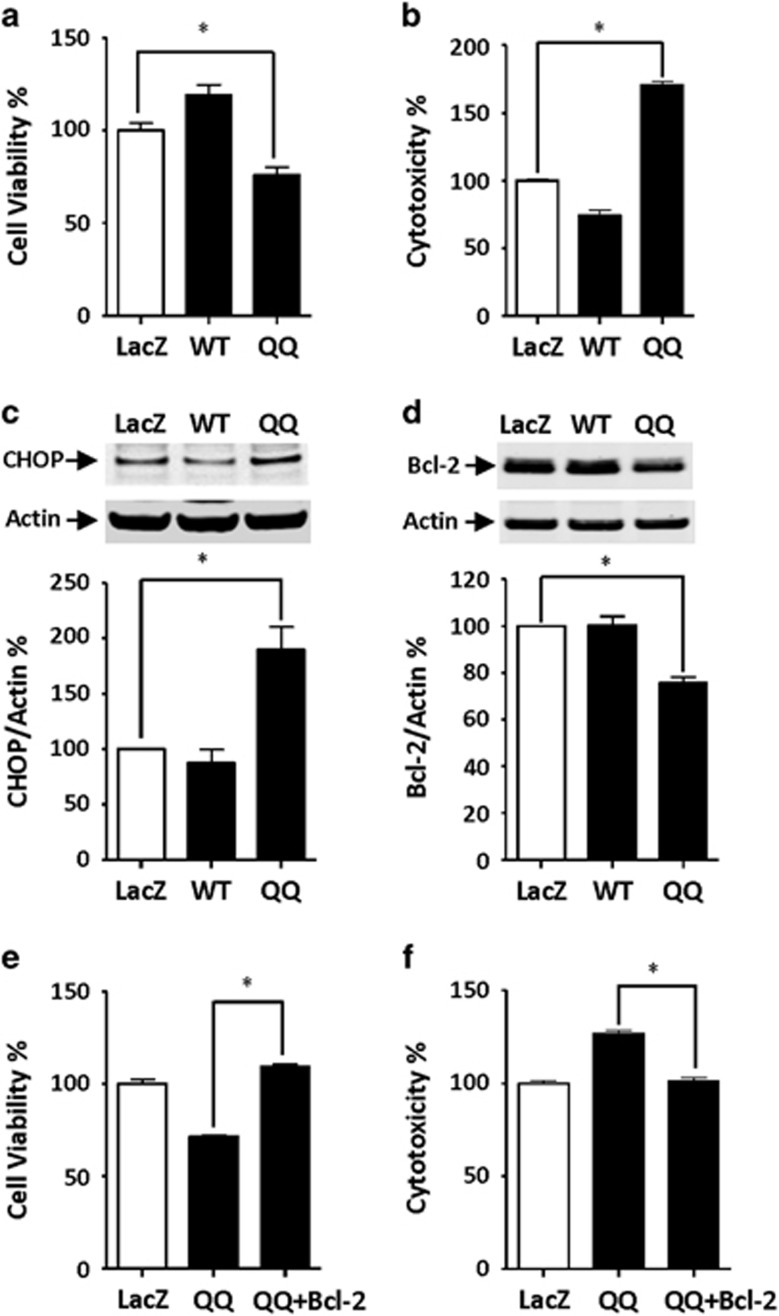
Effect of overexpression of carboxypeptidase E (CPE)-QQ on cellular function in hippocampal neurons. (**a**, **b**) Rat hippocampal neurons were transduced with adenovirus carrying LacZ (control), CPE-WT (WT) or CPE-QQ (QQ). Bar graphs show that CPE-QQ reduced cell viability ((**a**) WST-1 assay) and increased cell cytotoxicity ((**b**) Lactate dehydrogenase (LDH) assay) compared with cells expressing WT. One-way ANOVA analysis followed by Tukey test for WST-1 assay: F_(2,27)_=21.72, *P<0.05, *n*=10. One-way ANOVA analysis followed by Tukey test for LDH release assay: F_(2,12)_=338.6, *P<0.05, *n*=5. (**c**) Western blots of CHOP expression in hippocampal neurons expressing CPE-QQ and CPE-WT. Upper panel: blot showing CPE-QQ (QQ) increased the expression of CHOP compared with WT and LacZ. Lower panel: bar graphs showing quantification of western blots above. One-way ANOVA analysis followed by Tukey test: F_(2,6)_=16.26, *P<0.05, *n*=3. (**d**) Western blots of BCL-2 expression in hippocampal neurons expressing CPE-QQ and CPE-WT. Upper panel: blot showing CPE-QQ (QQ) down regulated the expression of BCL-2, an anti-apoptotic protein compared with WT and LacZ. Lower panel: bar graphs showing quantification of western blots above. One-way ANOVA analysis followed by Tukey test: F_(2,12)_=30.66, *P<0.05, *n*=3. (**e**, **f**) Transduction of BCL-2 in hippocampal neurons on cell viability and cytotoxicity. Bar graphs showing that transduction of BCL-2 in neurons expressing CPE-QQ (QQ) rescued the decreased cell viability (**e**) and increased cytotoxicity (**f**). One-way ANOVA analysis followed by Tukey test for WST-1 assay: F_(2,12)_=127.6, *P<0.05, *n*=5. One-way ANOVA analysis followed by Tukey test for LDH release assay: F_(2,12)_=79.5, *P<0.05, *n*=5.

We investigated whether CPE-QQ could cause cytotoxicity in hippocampal neurons. Figure 5a shows that expression of CPE-QQ in rat hippocampal neurons resulted in reduced cell viability as revealed by the WST assay. In addition, the lactate dehydrogenase assay indicates that hippocampal neurons expressing CPE-QQ show increased cytotoxicity compared with those expressing CPE-WT (Figure 5b). Analysis of CHOP shows an increase in expression in the neurons expressing CPE-QQ. This suggests that the decreased viability of neurons expressing CPE-QQ compared with neurons expressing CPE-WT (Figure 5c), is due to ER stress. Furthermore, the pro-survival mitochondrial protein, BCL-2, in hippocampal neurons expressing CPE-QQ, was decreased; indicating that the mechanism of action of CPE-QQ involves decreased expression of this protein, leading to apoptosis (Figure 5d). Overexpression of BCL-2 in hippocampal neurons expressing CPE-QQ rescued cell viability and cytotoxicity (Figures 5e and f).

### WT-CPE rescues cell viability in hippocampal neurons expressing CPE-QQ

**Figure 6:**
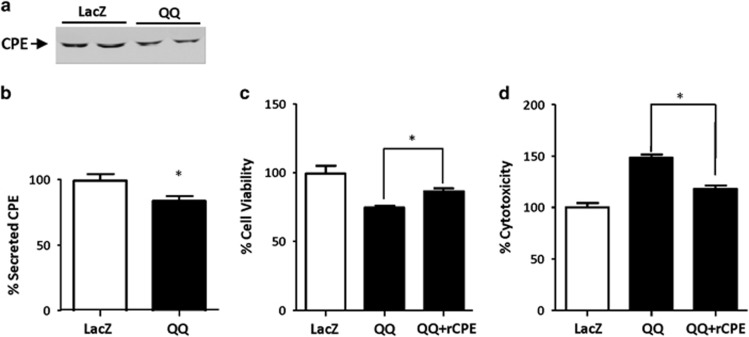
Carboxypeptidase E (CPE) rescues cell viability in CPE-QQ expressing neurons. (**a**, **b**) Rat hippocampal neurons were transduced with CPE-QQ and secretion medium collected. Western blot (**a**) and quantification (**b**, bar graphs) show expression of CPE-QQ reduces endogeneous CPE secretion. *t*-test, *P*<0.05, *n*=4. (**c**, **d**) Bar graphs show that incubation of the hippocampal neurons expressing CPE-QQ with 400 nM recombinant CPE (rCPE) rescued both their decreased cell viability (**c**) and increased cytotoxicity (**d**). One-way ANOVA analysis followed by Tukey test for WST-1 assay: F_(2,12)_=16.37, *P<0.05, *n*=5. One-way ANOVA analysis followed by Tukey test for lactate dehydrogenase release assay: F_(2,12)_=41.93, *P<0.05, *n*=5.

We determined if exogenously applied purified WT-CPE protein can rescue cell viability in hippocampal neurons expressing CPE-QQ. First, we showed that the secretion of endogenous WT-CPE from hippocampal neurons transduced with CPE-QQ was reduced compared with neurons transduced with LacZ (Figure 6a and b). Similar to that shown in Figure 5, the neurons transduced with CPE-QQ showed decreased cell viability and increased cytotoxicity. We then added purified WT-CPE protein (400 nm) to the medium to determine if that could rescue these defects. Figure 6c and d shows that exogenous WT-CPE protein rescued cell viability and cytotoxicity of the CPE-QQ neurons indicating that secreted CPE has an important role in mediating neuroprotection.

## Discussion

AD is commonly associated with psychiatric comorbidities, the most prevalent being depression.^31^ More than 50% of AD patients develop depression one year after diagnosis of the disease.^32^ However, the etiologies of this comorbidity remain poorly understood. A previous study has suggested a connection between CPE with AD. It showed aberrant accumulation of CPE in human AD plaques in different brain areas including the neocortex and hippocampus. Furthermore, CPE was aberrantly accumulated in degenerating neurites and activated astroglia of human AD plaques. In addition, amyloid-forming transgenic mice accurately recapitulate the aberrant CPE accumulation detected in AD dystrophic neurites.^33^ In this study, we investigated whether a novel mutation in the human CPE/NF-α1 gene (CPE-QQ) found in an AD patient could lead to comorbidity of dementia and depression. The likelihood that this mutation could cause memory deficits and depression linked to AD is very high as CPE/NF-α1 has been shown to have strong neuroprotective effects^27^ and prevents depression during chronic stress.^18^ Indeed, our study showed that transgenic mice overexpressing this CPE-QQ mutation exhibited memory loss and depressive-like behavior. However, they showed apparent normal metabolic function as they were not obese and had normal blood glucose levels, unlike the CPE/NF-α1 knock mouse^26^ and the CPE^ser202pro^ mutant mouse (CPE^*fat/fat*^).^24^

The CPE-QQ mutation described here consists of three adenosine inserts that results in the substitution of eight amino acids in the CPE-WT protein for night new amino acids in the first beta-pleated sheet after the pro-domain of the mutant CPE protein (Figures 1b and c). Interestingly, an EST sequence from pooled thalamic RNA of different humans (who died from brain trauma) with two adenosine and one cytosine insertions was also identified, that predicted the same core nine additional amino acids as in CPE-QQ, added to the CPE/NF-α1 protein, in addition to other amino acids upstream of this core (Supplementary Figure S7). The existence of a similar CPE mutant protein in humans suggests that the mutation is not unique to one individual and that such a mutant protein might also give rise to neurodegenerative disease and depression. In addition, the recent description of the first human with a truncating homozygous null mutation for CPE presenting with intellectual disability,^34^ further supports the importance of the CPE protein in cognitive function in humans. CPE-QQ-like mutations having the predicted nine amino acid insertions may be rare as a search of two human databases (1000 Genome and Esembl) have not revealed such a mutation. However, other human CPE mutations and SNPs exist, that could give rise to dementia and depression, given the link between CPE/NF-α1 and cognitive function.

Our biochemical studies demonstrated that the human CPE-QQ mutant protein is enzymatically inactive and therefore its role in prohormone processing is eliminated. However, the transgenic animals expressing this mutation did not show any weight gain or difference in levels of fasting glucose from WT animals indicating no apparent impairment of their metabolic functions. Unlike the CPE-knockout mouse,^26^ there is probably sufficient WT-CPE protein to perform the enzymatic prohormone processing functions in the CPE-QQ transgenic animals that also express CPE-WT. This is supported by our analysis that the levels of α-MSH in the hypothalamus (a peptide requiring enzymatic activity of CPE-WT to be generated) were unchanged in the transgenic mice compared with WT mice (Supplementary Figure S8A). In addition, our results also showed that the levels of orexin and GABA (which could affect mood and memory) were unchanged in the hypothalamus (Supplementary Figures S8B and C). We further analyzed MAP2 expression as a potential essential gene to determine if it could account for the observed phenotype as some brain regions showed reduced dendrites in CPE-QQ mice. Western blot analysis indicated that the total MAP2 expression did not change in CPE-QQ mice in hippocampus and hypothalamus (Supplementary Figures S8D and E), indicating that the loss of dendrites is not due to a decrease in expression of an essential gene such as MAP2.

We showed that the CPE-QQ mice exhibit memory deficits at 50 and 90 weeks of age, but with no impairment of spatial learning ability even at 90 weeks of age. In addition, they showed mild depressive-like behavior. Thus, these CPE-QQ mice exhibit comorbidity of loss of cognitive function and depression-like behavior. Analysis of the histopathology of the CPE-QQ mouse brains revealed that the dendrites were greatly reduced, indicating neurodegeneration, in the CA3 region and dentate gyrus of the hippocampus and the prefrontal cortex compared with WT mice, areas of the brain involved in cognitive function and depression.^35, 36^ In addition, we have analyzed several other brain regions including CA1 region of the hippocampus, parietal cortex and hypothalamus. The results indicated that the dendrites in these regions were normal in CPE-QQ mice (Supplementary Figures S9A–C), suggesting that changes of dendrites in CPE-QQ mice were restricted to certain regions in the brain. The CA3 region of the hippocampus normally expresses high levels of CPE and has been shown in the CPE-knockout mouse to be highly vulnerable to neurodegeneration during stress.^37^ Interestingly, we did not observe global loss of dendrites in the rest of the brain of the CPE-QQ mice such as CA1 region of the hippocampus, parietal cortex and hypothalamus (Supplementary Figures S9A–C). It is possible that other brain regions are less dependent on CPE for neuroprotection and sufficient protective effects may be provided by the endogenous WT-CPE in the transgenic mice. The loss of dendrites as observed in the CPE-QQ mice could lead to dementia, as dystrophic neurites is a major neuropathological characteristic of AD.^38^ Furthermore, dystrophic neurites have been found to be associated with the early stage extracellular NFTs in another type of dementia such as parkinsonism–dementia complex of Guam.^39^ Importantly, CPE-QQ mice showed abnormal hyperphosphorylation of tau at Ser^396^, which is specific for AD^29^ and phosphorylation of tau protein at sites Ser (396–404) is one of the earliest events in AD.^40^ This hyperphosphorylation has been shown to reduce affinity of microtubules, causing their destabilization and degeneration of neurons.^29^ Besides neurodegeneration, accumulating evidence indicates that altered adult neurogenesis have a critical role in the progression of AD^41^ and depression.^42^ We showed that these CPE-QQ mice have reduced neurogenesis in the subgranular zone of the hippocampus, a phenotype similar to the CPE-knockout mice, which exhibit severe depressive-like behavior.^18^ Thus, the impaired memory and depressive-like behavior in these CPE-QQ mice can be attributed to the neurodegeneration and diminished neurogenesis in these mutant mice, respectively. Altogether, this CPE mutation has a major impact on the function of the central nervous system, producing a pathological profile (Figures 2 and 3) in these CPE-QQ mice, with many characteristics of AD or other dementia, as evidenced by fewer dendrites in the hippocampus, memory deficits^43^ and hyperphosphorylation of tau.^44^ The decreased neurogenesis in the dentate gyrus likely accounts for the depressive phenotype in the mutant mice.^18, 45, 46^ The observed deficits in the CPE-QQ mice were unlikely to be caused by overexpression of CPE in abnormal brain regions as ICC did not show aberrant localization or excessive accumulation of CPE in the CPE-QQ mice compared with WT mice in several brain regions examined (Supplementary Figures S10A–D and Figures 11A–D). Moreover, western blot analyses indicate that CPE protein was not excessively overexpressed in the hippocampus (Supplementary Figure S10E), and hypothalamus (Supplementary Figure 11E) or pituitary (Supplementary Figure 11F) of CPE-QQ mice, although CPE-QQ mice did show a small but significant increase in CPE expression in hippocampus compared with WT mice. Interestingly, the deficits of dendrite outgrowth and hippocampal neurogenesis were not observed in younger CPE-QQ mice (11 week old), suggesting that CPE-QQ mice were more vulnerable to neurodegeneration during aging.

To understand the mechanism underlying the neurodegenerative phenotype of the CPE-QQ mutation, we carried out cell biological studies. CPE-QQ, when overexpressed in Neuro2a cells or hippocampal neurons, resulted in poor viability. This was attributed to the lack of trafficking of the mutant protein out of the ER causing ER stress as evidenced by the increase in CHOP in cells expressing the CPE-QQ mutation (Figures 4 and 5). In addition, it was found that this mutant caused diminished secretion of the endogenous WT-CPE protein (Figure 6a), suggesting that the mutant protein likely aggregated with the endogenous WT protein in the ER, preventing it from being trafficked through the secretory pathway for secretion. The protease inhibitor studies indicated that the accumulated mutant CPE protein in the ER is primarily degraded in proteasomes as the degradation was strongly inhibited by MG132; and possibly also through autophagy from the ER and final degradation in lysosomes as E64d, a lysosomal enzyme inhibitor, was effective at inhibiting the degradation. Indeed, degradation of aberrant proteins accumulated in the ER through both the proteosomal pathway and autophagy is quite common.^47^ We further showed that as a consequence of decreased secretion of CPE from hippocampal neurons when transduced with CPE-QQ (Figures 4 and 6), there was also a decrease in the expression of the mitochondria pro-survival (anti-apoptotic) protein, BCL-2. As it has been reported previously that CPE/NF-α1 acts as a signaling molecule to increase BCL-2 expression to protect neurons from cell death induced by oxidative stress,^27, 48^ insufficient CPE being secreted from cells expressing CPE-QQ likely accounts for the finding. Decreased neuronal cell viability in the CPE-QQ expressing cells was rescued by expressing BCL-2 or addition of exogenous CPE to the culture medium, indicating that diminished secreted CPE levels and BCL-2 expression were responsible for the poor cell viability of CPE-QQ neurons. Indeed, the poor neuronal viability phenotype is likely due to a combination of decreased CPE-WT activity, as we previously showed that CPE-knockout hippocampal neurons had less viability,^27^ as well as a toxic gain of function of CPE-QQ, which caused ER stress in the neurons.

In conclusion, cell biological studies indicate that the effect of the CPE-QQ mutation is accumulation in the ER of WT and mutant protein, resulting in ER stress and apoptosis of the neurons and decreased secretion of WT-CPE required for neuroprotection. At the physiological level, mice expressing CPE-QQ exhibit much fewer dendrites indicative of neurodegeneration and hyperphosphorylation of tau in the hippocampus and impaired memory, all hallmarks of AD. In addition, these mice showed depressive-like behavior likely due to diminished neurogenesis. Clearly, AD and other dementia are complex diseases with various etiologies involving genetic, epigenetic and environmental factors.^49, 50, 51^ The human mutation of the CPE/NFα-1 gene uncovered in this study has provided another unique potential genetic cause of AD that can give rise to comorbidity of dementia and depression. Moreover, this study reveals for the first time that humans with CPE/NF-α1 mutations could potentially be predisposed to dementia, as well as other cognitive function deficits.
