# Supplementary material for: A human carboxypeptidase E/NF-α1 gene mutation in an Alzheimer's disease patient leads to dementia and depression in mice
Source: Transl Psychiatry. 2016 Dec 6;6(12):e973–. doi: 10.1038/tp.2016.237 (PMC5315563; doi:10.1038/tp.2016.237)
Supplement: Supplementary Information [file tp2016237x1.pdf]

## Supplementary Methods

### Semi-quantitative PCR

RNA was extracted from the hippocampus of 50 week old WT and QQ CPE mice using the RNeasy mini kit (Qiagen), and quantified. First strand cDNAs were synthesized with 200 ng of RNA using Improm-II Reverse Transcription System (Promega). Semi-quantitative PCR was performed to detect CPE transcripts using GoTaq Green Master Mix (Promega). GAPDH was used for normalization. Primer sequences specific for the mouse CPE, fwd: 5'-AGGAAAGATCACAAGCTGACAATCGC -3', and CPE rev: 5'-GTAGCCTGGGTTGCCTCGGCTATGTA -3'; for the human CPE transgene, fwd: 5'-GGCAGCGTCTCACCCTGGTGAACTC-3', rev: 5'-TCCTTCGACGGACATGCTGGCATTC -3'. for GAPDH fwd: 5'- ACCACAGTCCATGCCATCAC-3', rev: 5'-TCCACCACCCTGTTGCTGTA -3'. One μl of cDNA was used for every reaction. PCR amplification was done for CPE (35 cycles) and GAPDH (25 cycles) at 94°C for 15 s, annealing at 60°C for 30 s, extension at 72°C for 30 s, and a final extension at 72°C for 10 min. Amplified PCR products were separated on 1.6 % agarose gels with Tris-borate EDTA buffer and stained with ethidium bromide. Gels were captured as digital images.

### Nissl Stain

The Nissl substance which is present exclusively in the somata of neuronal cells was detected by NeuroTrace green fluorescent Nissl stain (Invitrogen), following the instruction of the manufacturer. The images were taken with a fluorescence microscope (Nikon eclipse 80i) at 4X objective.

**ELISA**

Orexin and GABA levels were measured by ELISA kits from MyBioSource (San Diego, CA, USA), following the instruction of the manufacturer. α-MSH levels were measured by EIA kit from Phoenix Pharmaceuticals (Burlingame, CA, USA), following the instruction of the manufacturer.

**Figure S1. Semi-quantitative PCR of CPE transcripts.**

Top panel: Gels showing that the human CPE-QQ transcripts were detected in CPE-QQ mice (50 weeks old) but not in WT mice. Middle panel: WT and CPE-QQ mice expressed similar amounts of mouse CPE transcripts. Bottom panel: GAPDH as an internal control. n value: WT = 2, CPE-QQ = 2.

**Figure S2. Swim speed in 50 week old mice**

50 week old CPE-QQ mice and WT mice were subject to the Morris water maze test. Graph shows swim speed over 5 days. CPE-QQ and WT mice exhibited similar swim speed in the task. n vaule: WT = 12, CPE-QQ = 11.

**Figure S3**. **Memory deficits in 90 week old CPE-QQ mice.**

**A:** 90 week old CPE-QQ mice were subjected to the Morris water maze test. Graph shows the latency escape over 5 days. WT and QQ mice exhibited a normal acquisition curve in the water

maze task. **B:** Bar graphs show time spent by the mice in each quadrant during the probe test. WT mice spent more time in the target quadrant than QQ mice. (T: target quadrant, L: left quadrant, O: opposite quadrant, R: right quadrant.) *t*-test of target quadrant: $p < 0.05$, n value: WT = 6, CPE-QQ = 7.

**Figure S4. Sucrose preference test in 50 week old CPE-QQ mice**

Bar graphs show CPE-QQ mice had the same preference for sucrose intake compared to WT mice. t-test, $p > 0.05$. n value: WT = 8, CPE-QQ = 9.

**Figure S5. Histology of CPE-QQ mouse hippocampus and pre-frontal cortex**.

A: Fluorescent microscope (4X) images of Nissl staining showing that 50 wk old CPE-QQ mice appear to have similar neuronal architecture in the hippocampus compared to WT controls. Scale bar: 200 µm.

B: Confocal images (20X) of the dentate gyrus showing reduced numbers of dendrites in the hilus of CPE-QQ mice (50 weeks old) compared to the WT mice. Scale bar: 50 µm.

C: Confocal images of the prefrontal cortex showing reduced dendrite outgrowth in the CPE-QQ mice (50 week old) compared to the WT mice. Top panel: diagram of prefrontal cortex. Middle panels: 10X confocal images, scale bar: 100 µm. Bottom panels: 20X confocal images, scale bar: 50 µm. n value: WT = 4, CPE-QQ = 4.

**Figure S6**: **Histology of CPE-QQ mice at 11 weeks.**

**A:** Confocal images (20X) of the dentate gyrus showing no difference of dendrite outgrowth in the hilus of CPE-QQ mice (11 week old) compared to the WT mice. Scale bar: 50 µm.

**B:** Confocal images (20X) showed that 11 week old QQ mice had similar doublecortin staining in the dentate gyrus (DG) compared to WT mice, indicating that younger QQ mice did not have deficits in hippocampal neurogenesis. Scale bar: 50 µm.

**C:** Quantification of doublecortin positive cells in DG. *t*-test, $p > 0.05$. n value: WT = 4, CPE-QQ = 4.

**Figure S7.** Nucleotide and predicted amino acid sequence of an additional EST sequence found from thalami of humans. **A**: Nucleotide and **B**: amino acid alignment of human WT CPE and the human EST sequence from the thalamus (Genebank ID# DA397828). Inserted nucleotides (**A**) or amino acids (**B**) in the mutant CPE sequence are shown in red.

**Figure S8**: **CPE processing activity in hypothalamus and hippocampus of CPE-QQ mice.**

**A:** Bar graphs show α-MSH levels in hypothalamus in 90 week old CPE-QQ and WT mice assayed by EIA, *t*-test, n = 6, $p > 0.05$.

**B:** Bar graphs show Orexin levels in hypothalamus in 50 week old CPE-QQ and WT mice assayed by ELISA, *t*-test, n = 4, $p > 0.05$.

**C:** Bar graphs show GABA levels in hypothalamus in 50 week old CPE-QQ and WT mice assayed by ELISA, *t*-test, $n = 4$, $p > 0.05$.

**D:** Bar graphs show MAP2 levels in hypothalamus in 50 week old CPE-QQ and WT mice assayed by western blot. HT, hypothalamus. t- test, $n = 4$, $p > 0.05$.

**E:** Bar graphs show MAP2 levels in hippocampus in 50 week old CPE-QQ and WT mice assayed by western blot. HP, hippocampus. t-test, $n = 4$, $p>0.05$.

**Figure S9**: **Histology of CPE-QQ mice at 50 weeks.**

Confocal images (20X) of the CA1 region of hippocampus (A), parietal cortex (B) and hypothalamus (C) showing no difference of dendrite outgrowth (MAP2 staining) in CPE-QQ mice (50 week old) compared to the WT mice . Scale bar: 50 µm. n = 4.

**Figure S10**: **CPE expression in the hippocampus of 50 week old WT and CPE-QQ mice.**

**A, B:** Confocal images (10X) showing CPE expression in the CA3 **(A)** and CA1 **(B)** region of hippocampus. Scale bar: 100 µm. n = 4.

**C, D:** Confocal images (20X) showing CPE expression in the CA3 (C) and CA1 (D) region of hippocampus. Scale bar: 50 µm. n = 4.

**E:** Western blot showing CPE levels in hippocampus. HP, hippocampus. t-test, $p < 0.05$. n = 3.

**Figure S11: CPE expression in the hypothalamus, neocortex and pituitary of 50 week old WT and CPE-QQ mice.**

**A, B:** Confocal images (10X) showing CPE expression in the hypothalamus **(A)** and neocortex **(B)**. Scale bar: 100 µm. n = 4.

**C, D:** Confocal images (20X) showing CPE expression in the hypothalamus **(C)** and neocortex **(D).** Scale bar: 50 µm. n = 4.

**E:** Western blot showing CPE levels in hypothalamus. HT, hypothalamus. t-test, $p > 0.05$. n = 4.

**F:** Western blot showing CPE levels in pituitary. t-test, $p > 0.05$. n = 3.

**Fig. S1**

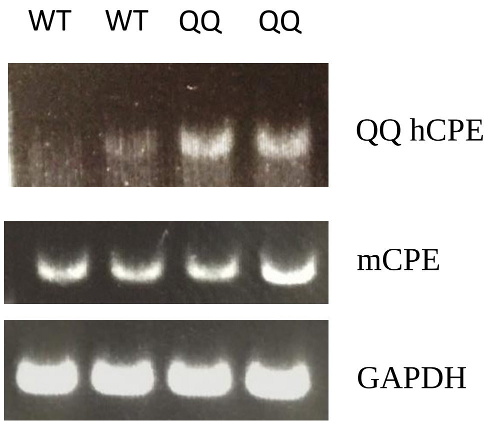

**Fig. S2**

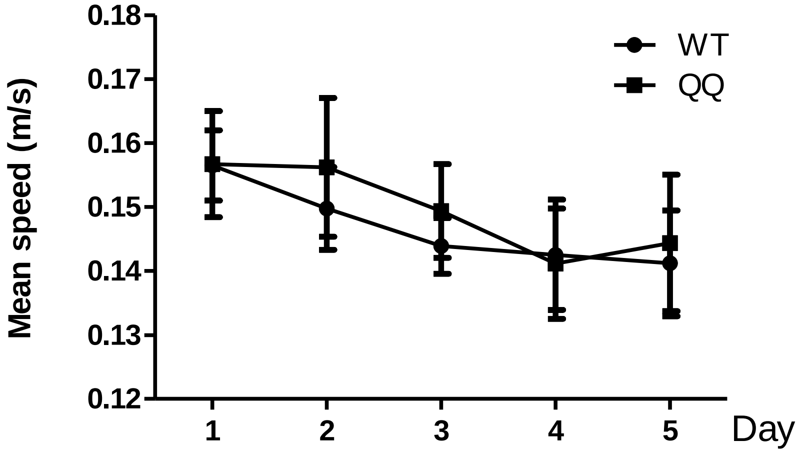

**Fig. S3**

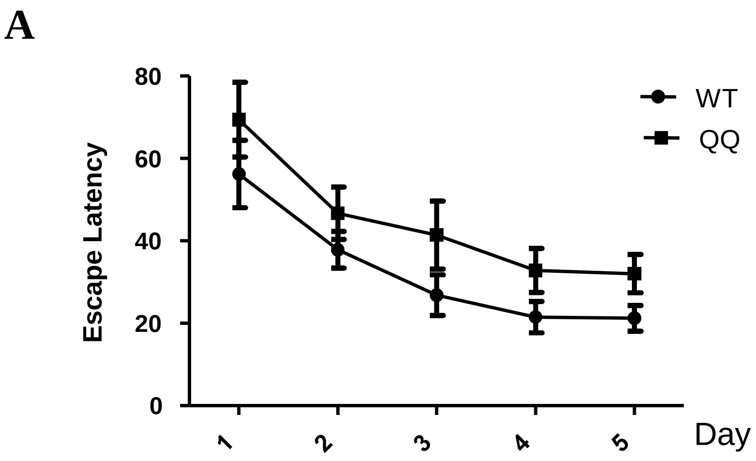

A
Escape Latency
80
60
40
20
0
1
2
3
4
5
Day
WT
QQ


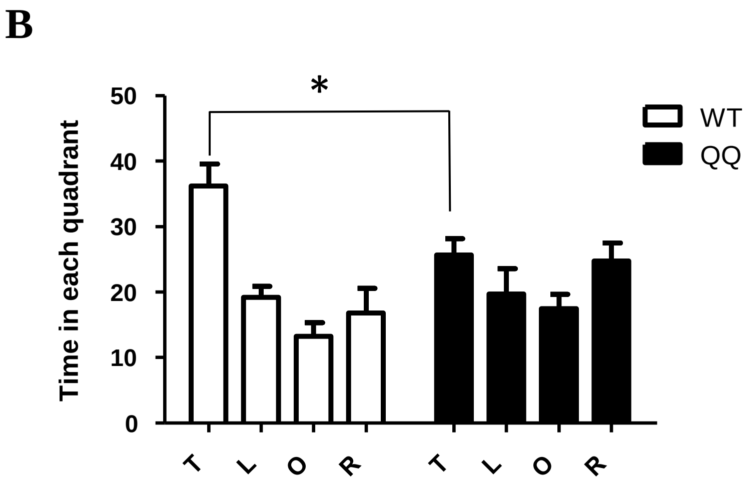

B
*
Time in each quadrant
50
40
30
20
10
0
T
L
O
R
T
L
O
R
WT
QQ

# Fig. S4

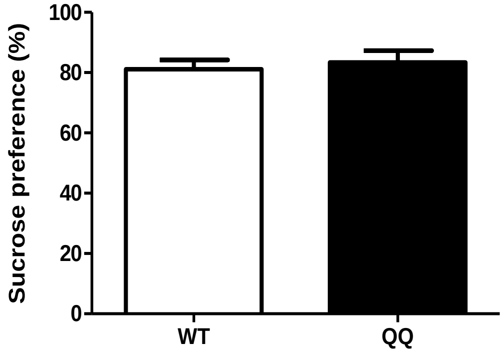

# Fig. S5

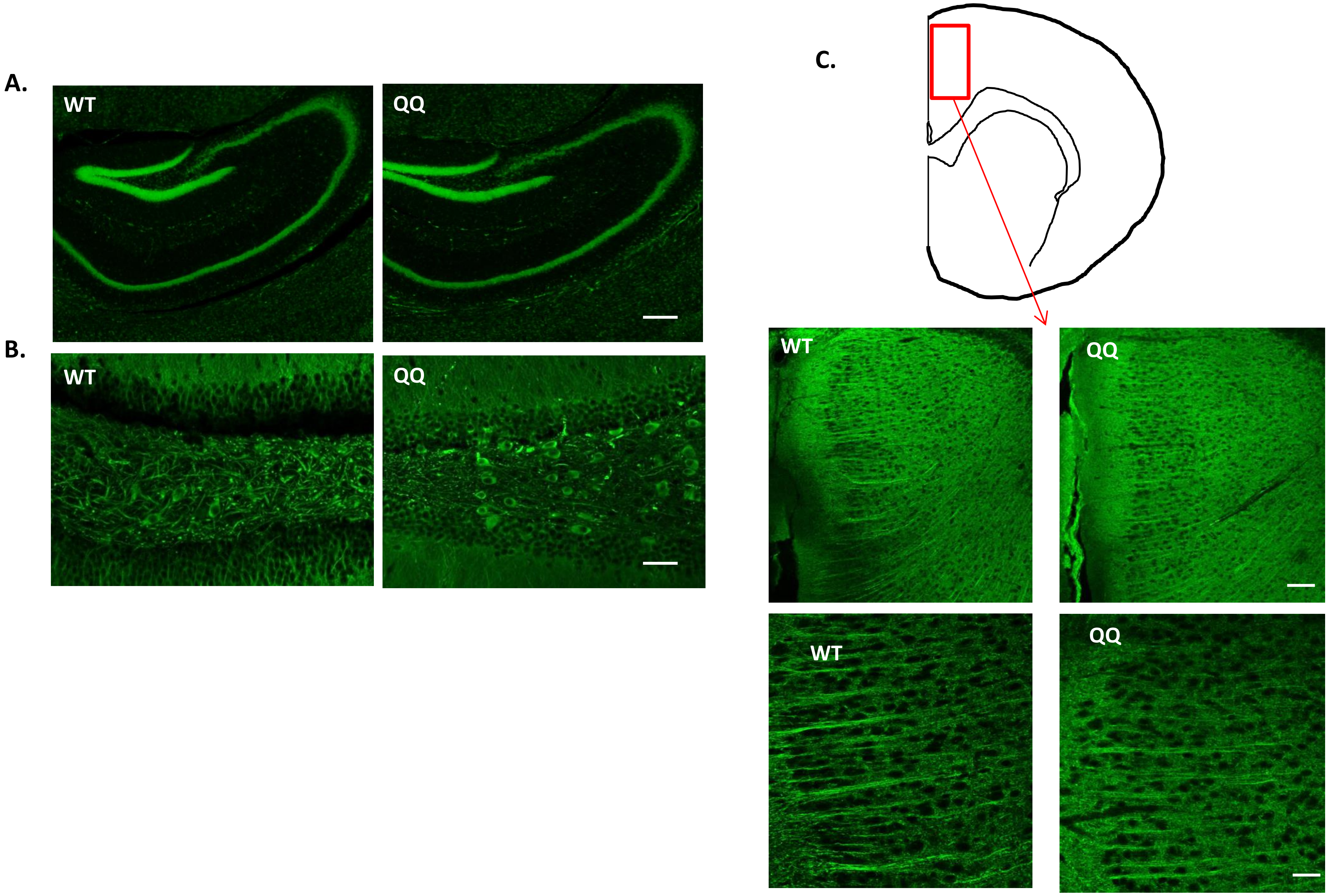

**Fig. S6**

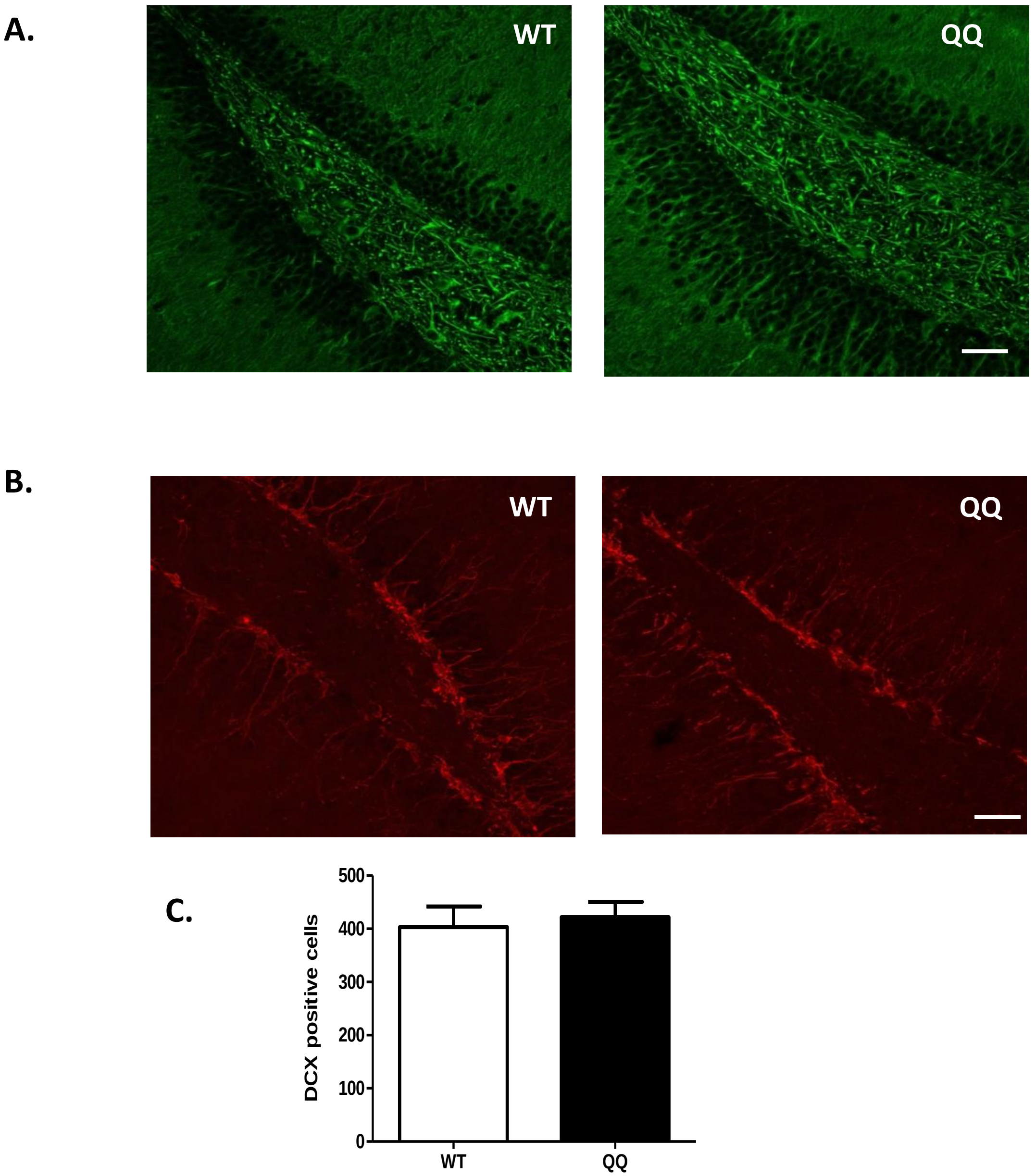

# Fig. S7

**A.**

```
WT        CATCTCCTTCGAGTACCACCGCTACCCCGAGCTGCGCGAGGC-GCTCGTGTCCGTGTGGC
Thalamus  CATCTCCTTCGAGTACCACCGCTACCCCGAGCTGCGCGAGGCAGCTCGTGTCCGTGTGGC

WT        TGCAGTGCACCGCCATCAGCAGGATTTACAC-GGTGGGGCGCAGC-TTCGAGGGCCGG
Thalamus  TGCAGTGCACCGCCATCAGCAGGATTTACACAGGTGGGGCGCAGCCTTCGAGGGCCGG
```

**B.**

```
WT        I S F E Y H R Y P E L R E A L V S V W L Q C T - A I S R I Y T V G R S F E G R
Thalamus  I S F E Y H R Y P E L R E A A R V R V A A V H R H Q Q D L H R W G A A F E G R
```

Fig. S8

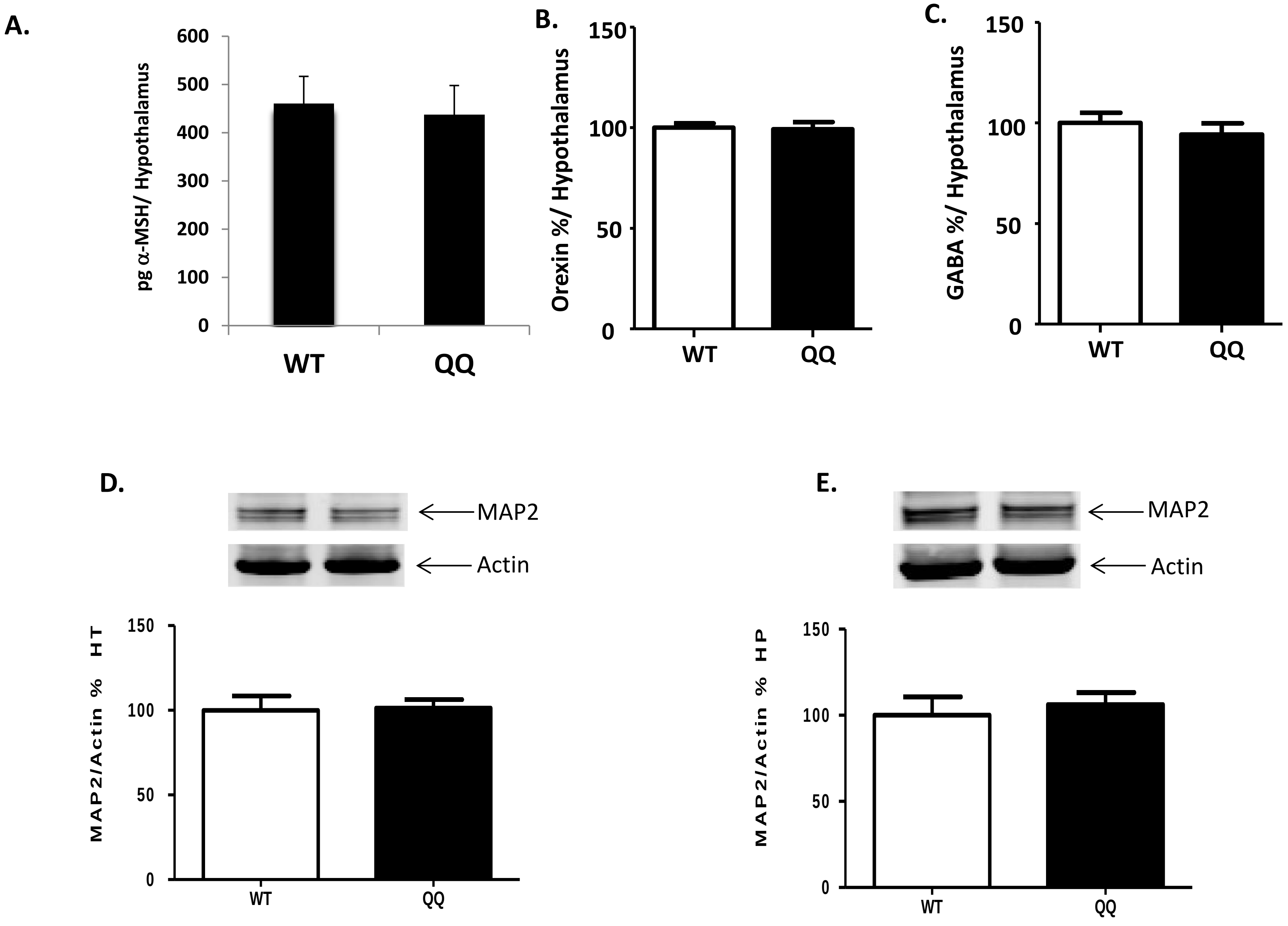
A.
pg α-MSH/ Hypothalamus
600
500
400
300
200
100
0
WT
QQ
B.
Orexin %/ Hypothalamus
150
100
50
0
WT
QQ
C.
GABA %/ Hypothalamus
150
100
50
0
WT
QQ
D.
MAP2
Actin
MAP2/Actin % HT
150
100
50
0
WT
QQ
E.
MAP2
Actin
MAP2/Actin % HP
150
100
50
0
WT
QQ

# Fig. S9

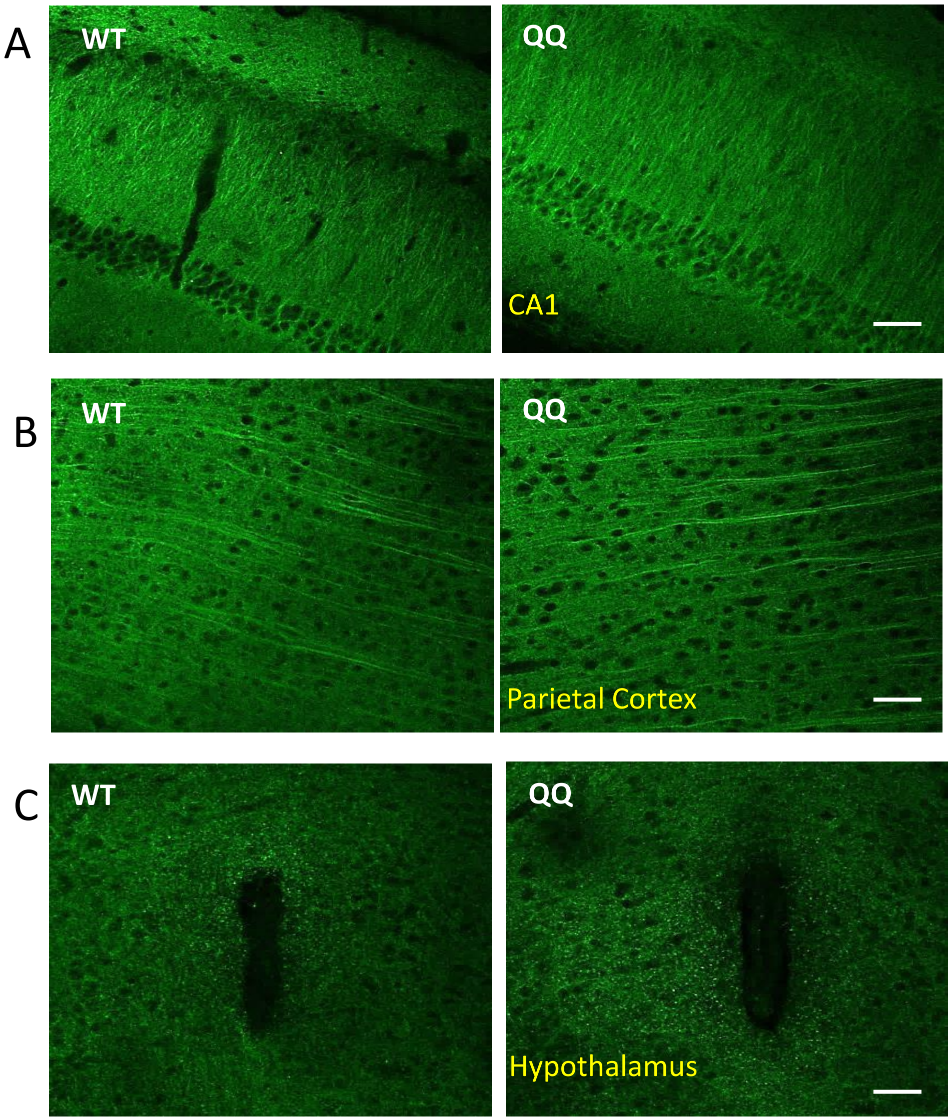

Fig. S10

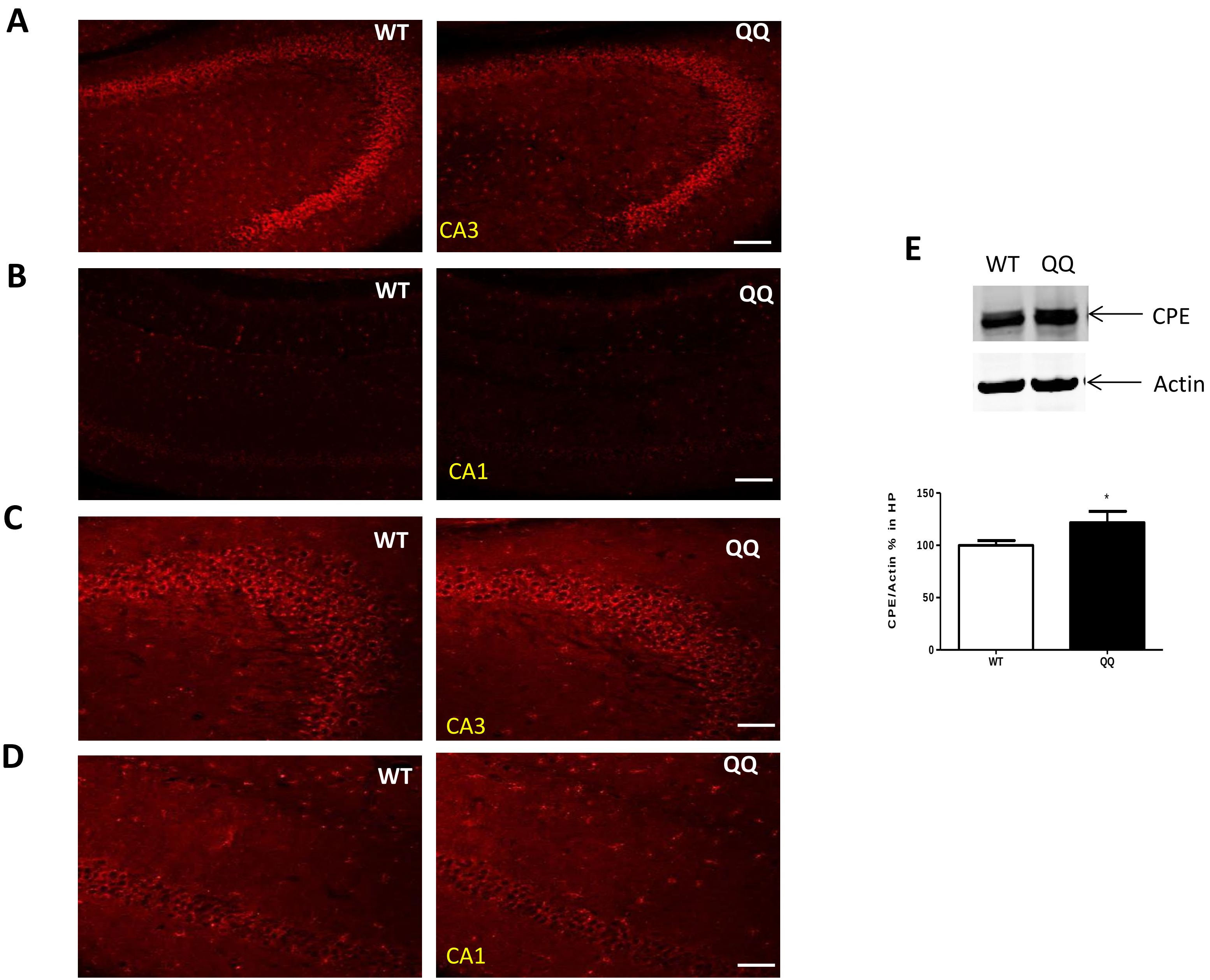
A
WT
QQ
CA3
B
WT
QQ
CA1
C
WT
QQ
CA3
D
WT
QQ
CA1
E
WT
QQ
CPE
Actin
CPE/Actin % in HP
150
100
50
0
*
WT
QQ

Fig. S11

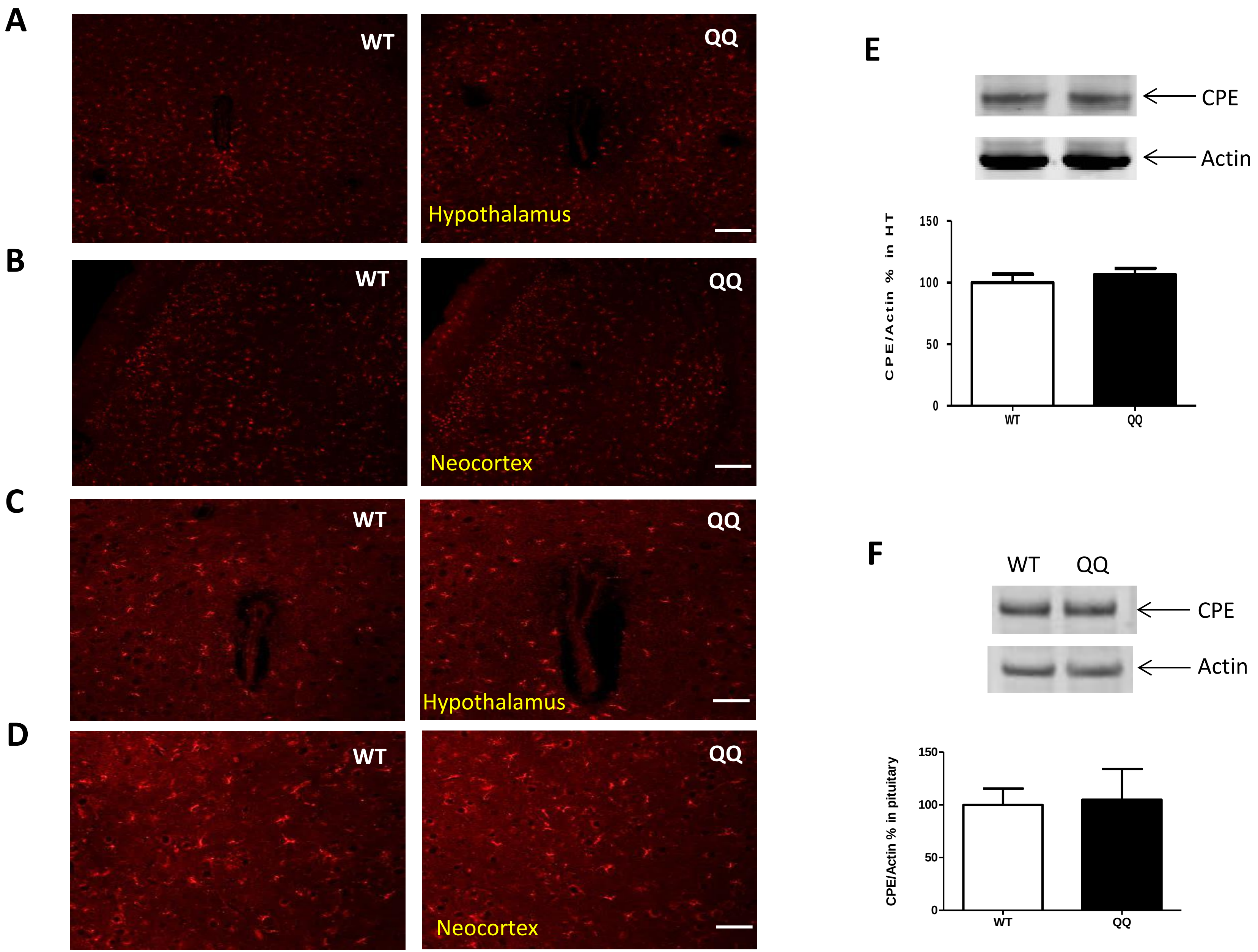
A
WT
QQ
Hypothalamus
B
WT
QQ
Neocortex
C
WT
QQ
Hypothalamus
D
WT
QQ
Neocortex
E
CPE
Actin
CPE/Actin % in HT
150
100
50
0
WT
QQ
F
WT
QQ
CPE
Actin
CPE/Actin % in pituitary
150
100
50
0
WT
QQ
